# Febrile children with comorbidities at the emergency department — a multicentre observational study

**DOI:** 10.1007/s00431-022-04552-2

**Published:** 2022-07-07

**Authors:** Dorine M. Borensztajn, Nienke N. Hagedoorn, Enitan D. Carrol, Ulrich von Both, Marieke Emonts, Michiel van der Flier, Ronald de Groot, Jethro Herberg, Benno Kohlmaier, Michael Levin, Emma Lim, Ian K. Maconochie, Federico Martinon-Torres, Ruud G. Nijman, Marko Pokorn, Irene Rivero-Calle, Maria Tsolia, Fabian J. S. van der Velden, Clementien Vermont, Dace Zavadska, Werner Zenz, Joany M. Zachariasse, Henriette A. Moll

**Affiliations:** 1 Department of General Paediatrics, Erasmus MC-Sophia Children’s Hospital, P.O. Box 2060, 3000 CB Rotterdam, The Netherlands; 2 Institute of Infection, Veterinary and Ecological Sciences, University of Liverpool, Liverpool, UK; 3 Department of Infectious Diseases, Alder Hey Children’s NHS Foundation Trust, Liverpool, UK; 4 Liverpool Health Partners, Liverpool, UK; 5 Division of Paediatric Infectious Diseases, Dr. Von Hauner Children’s Hospital, University Hospital, Ludwig-Maximilians-University (LMU), Munich, Germany; 6 DZIF, German Centre for Infection Research, Partner Site Munich, Munich, Germany; 7 Great North Children’s Hospital, Paediatric Immunology, Infectious Diseases & Allergy, Newcastle Upon Tyne Hospitals NHS Foundation Trust, Newcastle upon Tyne, UK; 8 Translational and Clinical Research Institute, Newcastle University, Newcastle upon Tyne, UK; 9 NIHR Newcastle Biomedical Research Centre Based at Newcastle Upon Tyne Hospitals NHS Trust and Newcastle University, Newcastle upon Tyne, UK; 10 Department of Paediatric Infectious Diseases and Immunology, Amalia Children’s Hospital, Radboudumc, Nijmegen, The Netherlands; 11 Department of Paediatric Infectious Diseases and Immunology, Wilhelmina Children’s Hospital, University Medical Centre Utrecht, Utrecht, The Netherlands; 12 Section Paediatric Infectious Diseases, Laboratory of Medical Immunology, Department of Laboratory Medicine, Radboud Institute for Molecular Sciences, Radboud University Medical Centre, Nijmegen, The Netherlands; 13 Radboud Center for Infectious Diseases, Radboudumc, 6525 GA Nijmegen The Netherlands; 14 Section of Paediatric Infectious Diseases, Imperial College of Science, Technology and Medicine, London, UK; 15 Department of General Paediatrics, Medical University of Graz, Graz, Austria; 16 Genetics, Vaccines, Infections and Pediatrics Research Group (GENVIP), Hospital Clínico Universitario de Santiago de Compostela, Santiago de Compostela, Spain; 17 Department of Infectious Diseases, University Medical Centre Ljubljana, Univerzitetni Klinični Center, Ljubljana, Slovenia; 18 Second Department of Paediatrics, National and Kapodistrian University of Athens, P. and A. Kyriakou Children’s Hospital, Athens, Greece; 19 Department of Pediatric Infectious Diseases & Immunology, Erasmus MC-Sophia Children’s Hospital, Rotterdam, The Netherlands; 20 Department of Pediatrics, Children Clinical University Hospital, Rīgas Stradiņa universitāte, Riga, Latvia

**Keywords:** Chronic disease, Comorbidity, Fever, Infectious diseases, Emergency care

## Abstract

We aimed to describe characteristics and management of children with comorbidities attending European emergency departments (EDs) with fever. MOFICHE (Management and Outcome of Fever in children in Europe) is a prospective multicentre study (12 European EDs, 8 countries). Febrile children with comorbidities were compared to those without in terms of patient characteristics, markers of disease severity, management, and diagnosis. Comorbidity was defined as a chronic underlying condition that is expected to last > 1 year. We performed multivariable logistic regression analysis, displaying adjusted odds ratios (aOR), adjusting for patient characteristics. We included 38,110 patients, of whom 5906 (16%) had comorbidities. Most common comorbidities were pulmonary, neurologic, or prematurity. Patients with comorbidities more often were ill appearing (20 versus 16%, *p* < 0.001), had an ED-Paediatric Early Warning Score of > 15 (22 versus 12%, *p* < 0.001), or a C-reactive protein > 60 mg/l (aOR 1.4 (95%CI 1.3–1.6)). They more often required life-saving interventions (aOR 2.7, 95% CI 2.2–3.3), were treated with intravenous antibiotics (aOR 2.3, 95%CI 2.1–2.5), and were admitted to the ward (aOR 2.2, 95%CI 2.1–2.4) or paediatric intensive care unit (PICU) (aOR 5.5, 95% CI 3.8–7.9). They were more often diagnosed with serious bacterial infections (aOR 1.8, 95%CI 1.7–2.0), including sepsis/meningitis (aOR 4.6, 95%CI 3.2–6.7). Children most at risk for sepsis/meningitis were children with malignancy/immunodeficiency (aOR 14.5, 8.5–24.8), while children with psychomotor delay/neurological disease were most at risk for life-saving interventions (aOR 5.3, 4.1–6.9) or PICU admission (aOR 9.7, 6.1–15.5).

*Conclusions:* Our data show how children with comorbidities are a population at risk, as they more often are diagnosed with bacterial infections and more often require PICU admission and life-saving interventions.

**Table:** 

**What is Known:** *• While children with comorbidity constitute a large part of ED frequent flyers, they are often excluded from studies.*	
**What is New:** *• Children with comorbidities in general are more ill upon presentation than children without comorbidities.* *• Children with comorbidities form a heterogeneous group; specific subgroups have an increased risk for invasive bacterial infections, while others have an increased risk of invasive interventions such as PICU admission, regardless of the cause of the fever.*

**Supplementary information:**

The online version contains supplementary material available at 10.1007/s00431-022-04552-2.

## Introduction

There is an important health paradox regarding children with comorbidities attending the emergency department (ED). On one hand, these children form an increasing group [1]; constitute a large part of the so-called “ED frequent flyers” [2, 3]; have an increased risk for invasive bacterial infections (sepsis/meningitis) as well as serious bacterial infections, such as urinary tract infections, pneumonia, or bone and joint infections [4–7]; and use a larger amount of ED and hospital resources [8, 9], yet on the other hand are very often excluded from studies [10–13]. When research does include children with comorbidities, it usually focuses on specific types of comorbidities, such as febrile neutropenia [14]. The most compelling evidence that shows how children with comorbidities form a very fragile population comes from a recent key paper, showing half of the infection-related deaths in the UK occur in children with comorbidities [15].

However, no details were provided regarding type of comorbidities or presenting problems. Our aim was to address this knowledge gap, by assessing presenting signs and symptoms, clinical management, and cause of infection of febrile children with comorbidities attending the ED in a large European cohort.

## Materials and methods

### Study design

This study is part of the MOFICHE (Management and Outcome of Febrile children in Europe) study, which is embedded in the PERFORM (Personalized Risk assessment in Febrile illness to Optimise Real-life Management across the European Union) study. The MOFICHE study is an observational multicentre study that evaluates the clinical management and outcome of febrile children in Europe using routinely collected data [16].

### Ethics statement

The study was approved by the ethical committees of all the participating hospitals, and no informed consent was needed for this study: Austria (Ethikkommission Medizinische Universitat Graz, ID:28-518ex15/16), Germany (Ethikkommission Bei Der LMU München, ID:699–16), Greece (Ethics committee, ID:9683/18.07.2016), Latvia (Centrala medicinas etikas komiteja, ID:14.07.201.6.No. Il16-07–14), Slovenia (Republic of Slovenia National Medical Ethics Committee, ID:0120–483/2016–3), Spain (Comité Autonómico de Ética de la Investigación de Galicia, ID:2016/331), The Netherlands (Commissie Mensgebonden onderzoek, ID:NL58103.091.16), UK (Ethics Committee, ID:16/LO/1684, IRAS application no. 209035, Confidentiality advisory group reference: 16/CAG/0136).

In all the participating UK settings, an additional opt-out mechanism was in place.

### Study population and setting

Twelve EDs from eight different European countries (Austria, Germany, Greece, Latvia, the Netherlands (*n* = 3), Spain, Slovenia, and the UK (*n* = 3)) participated in the study. Participating hospitals were either tertiary university hospitals or large teaching hospitals (Appendix 1). Data were collected for at least 1 year, between January 2017 and April 2018. Inclusion criteria were children aged 0 months to 18 years presenting with fever to the ED (temperature ≥ 38.0 °C) or a history of fever in the previous 72 h.

### Data collection

Data were obtained from patient records and entered into an electronic case report form. Data included general patient characteristics (age, sex, comorbidity, previous medical care, time of arrival, referral (self, primary care physician, Emergency Medical Services, or other), triage urgency, vital signs, and presence of “red traffic light” alarming signs for identifying risk of serious illness (National Institute for Health and Care Excellence (NICE) guideline on fever [17]. These alarming signs include level of consciousness, ill-appearance, increased work of breathing, age < 3 months, non-blanching rash, meningeal signs, status epilepticus, and focal neurological signs.

Data collection ranged from a 1 week per month sample to all visits, depending on the number of ED visits per hospital (Appendix 1).

### Definitions

The presence of comorbidity and type of comorbidity (e.g., organ system involved) were taken directly from the patient’s chart. Comorbidity was pre-defined as a chronic underlying condition that is expected to last at least one year. During data analysis, comorbidity was classified as either simple or complex. Complex comorbidity was defined as comorbidity that affects two or more body system, malignancy, or a progressive condition [18–20]. Children with malignancy and immunodeficiency were analysed together, as were children with neurologic disease and psychomotor delay.

Vital signs were classified as abnormal according to APLS reference ranges.

ED-Paediatric Early Warning Score (ED-PEWS) were calculated based on the PEWS specifically developed and validated for the ED by Zachariasse et al. (age, vital signs, capillary refill time, level of consciousness, and work of breathing combined into an ED- PEWS score) [21].

Triage categories were grouped together into low urgency (non-urgent, standard) and high urgency (urgent, very urgent, and immediate).

A previous ED visit was defined as a visit to either the same or a different ED in the previous 5 days. Duration of fever was defined as the duration of fever upon presentation at the ED.

Immediate life-saving interventions (ILSI) were categorized into the following categories: airway and breathing support, electrical therapy, emergency procedures, hemodynamic support, and emergency medications (Appendix 2) [22, 23].

Focus of infection was categorized as upper respiratory tract infection (otitis media, tonsillitis/pharyngitis, other), lower respiratory tract infection (LRTI), gastro-intestinal tract and surgical abdomen, urinary tract, skin, musculoskeletal, sepsis, meningitis/central nervous system, flulike illness, childhood exanthemas, inflammatory syndromes, undifferentiated fever, or other [16].

The consortium developed a consensus-based flowchart [16, 24] combining all available clinical data and test results. A more detailed description was published by Hagedoorn et al. [16] This flowchart was used to classify the presumed cause of infection for each patient visit (Appendix 3), depending on clinical signs, C-reactive protein (CRP), and microbiological tests (bacterial cultures, viral or bacterial PCR).

Serious bacterial infections were defined as “definite/probable bacterial” with a focus of infection from the gastro-intestinal tract, LRTI, urinary tract, bone and joints, central nervous system, or sepsis [22]. Invasive bacterial infection (sepsis/meningitis) was defined as a focus from the central nervous system or sepsis and “definite/probable bacterial” from the consensus-based flowchart [25].

### Data quality and missing data

The use of a digital training module, which included the clarification of the National Institute for Health and Care Excellence alarming signs, allowed optimization and standardization of the clinical assessment and data collection processes. Universal guidance on entering the standardized data was issued prior to commencement of the study period. Furthermore, monthly teleconferences and biannual meetings were organized and quarterly reports of data quality were discussed with all partners.

Missing determinants were handled by using multiple imputation. Imputation was performed by using the MICE package in R, version 3.4. SPSS version 25 was used for the analysis of the data. This imputation process resulted in twenty databases on which statistical analysis was performed and pooled for a final result.

### Data analysis

We performed descriptive analyses for general patient characteristics, vital signs, ED-PEWS and presence of National Institute for Health and Care Excellence alarming signs, clinical management (diagnostic tests, intravenous antibiotics, oxygen therapy, immediate life-saving interventions), disposition (discharge, hospital admission, Paediatric Intensive Care Unit (PICU) admission), and diagnosis (focus of infection, viral or bacterial disease). Characteristics of children with and without comorbidities were compared using chi-squared-tests and Mann–Whitney *U* tests. Results were deemed significant with a *p*-value < 0.05.

We analysed differences in management, disposition, and presumed cause of infection for children with and without comorbidities by multivariable logistic regression adjusted for ED of presentation and general patient characteristics (sex, duration of fever, previous medical care, time of arrival, and mode of referral).

## Results

After excluding 370 (1.0%) patients with missing data regarding comorbidities, 38,110 patients were left for analysis. In total, 5906 patients had comorbidities (16%, range between EDs 5.3 and 62%) of whom 1678 (28%, range 8.6–60%) were classified as complex comorbidities (Table 2). The most common types of comorbidities were pulmonary, neurologic/psychomotor delay, prematurity (gestational age < 37 weeks), urology/nephrology, cardiac, and malignancy/immunodeficiency (Table 1). Details regarding missing variables are provided in Table 2.

**Table 1:** Types of comorbidities

**Level of complexity, *n* = 38,110**	
None	32,204 (84.5)
Non-complex comorbidity	4228 (11.1)
Complex comorbidity	1678 (4.4)
Type of comorbidity, *n* = 5906*	
Pulmonary	1414 (23.9)
Neurologic	1108 (18.8)
Prematurity	1024 (17.3)
Psychomotor delay	809 (13.7)
Urology/nephrology	712 (12.1)
Cardiac	623 (10.5)
Immunodeficiency	508 (8.6)
Malignancy	297 (5.0)
Gastrointestinal	227 (3.8)
Hematologic	298 (5.0)
Metabolic	222 (3.8)
Other	460 (7.8)

^*^Multiple categories possible

**Table 2:** Differences in patient characteristics between children with and without comorbidities (*n* = 38,110)

	**No comorbidity** ***N* = 32,204** ***N* (%)**	**Comorbidity (any)**^**a**^ ***N* = 5906** ***N* (%)**	**No comorbidity versus any comorbidity** ***P***	**Non-complex comorbidity**^**a**^ ***N* = 4228** ***N* (%)**	**Complex comorbidity**^**a**^ ***N* = 1678** ***N* (%)**
**Male**	17,424 (54)	3532 (59%)	< 0.001	2477 (59)	1012 (60)
**Age in years, median (IQR)**	2.6 (1.3–5.3)	3.7 (1.6–7.7)	< 0.001	3.5 (1.5–7.1)	4.4 (2.0–9.2)
**Duration of fever**			< 0.001		
< 24 h	10,528 (35)	2301 (44)		1573 (41)	728 (50)
24–48 h	9935 (33)	1463 (28)		1101 (29)	362 (25)
> 48 h	9722 (33)	1505 (29)		1123 (30)	382 (26)
**Previous ED visit**	2427 (7.5)	688 (12%)	< 0.001	444 (10)	244 (15)
**Referral**			< 0.001		
Self	18,377 (59)	2656 (47)		2038 (51)	618 (39)
GP/private paediatrician	5373 (17)	984 (17)		759 (19)	225 (14)
Emergency medical service	4826 (15)	718 (13)		538 (13)	180 (11)
Other	2773 (8.8)	1250 (22)		698 (17)	552 (35)
**Triage urgency**			< 0.001		
High	10,071 (32)	2988 (53)		1881 (46)	1107 (69)
**Vital signs**^**b**^** and PEWS**					
Tachycardia	7736 (24)	1708 (29)	< 0.001	1135 (27)	573 (34)
Tachypnoea	4377 (14)	1200 (20)	< 0.001	804 (19.0)	396 (24)
Hypoxia, oxygen saturation < 95%	572 (1.8)	273 (4.6)	< 0.001	176 (4.2)	97 (5.8)
Prolonged capillary refill ≥ 3 s	336 (1.2)	86 (1.7)	< 0.05	51 (1.4)	35 (2.6)
ED-PEWS < 6	6834 (21)	978 (17)	< 0.001	742 (17)	236 (14)
ED-PEWS ≥ 15	3962 (12)	1299 (22)	< 0.001	858 (20)	441 (27)
**NICE “red traffic lights” (alarming signs)**					
Ill appearance	4918 (16)	1063 (20)	< 0.001	747 (19)	316 (22)
Increased work of breathing	2343 (8.3)	870 (18)	< 0.001	572 (16)	298 (22)
Rash: petechiae/non-blanching	968 (3.0)	130 (2.2)	< 0.05	99 (2.3)	31 (1.8)
Decreased consciousness	123 (0.4)	77 (1.3)	< 0.001	37 (0.9)	40 (2.5)
Meningeal signs	109 (0.4)	27 (0.5)	0.064	17 (0.4)	10 (0.7)
Status epilepticus	33 (0.1)	31 (0.5)	< 0.001	12 (0.3)	19 (1.2)
Focal neurology	72 (0.2)	58 (1.1)	< 0.001	25 (0.6)	33 (2.4)
**Disposition and therapy**					
Admission	7499 (23)	2136 (36)	< 0.001	1360 (32)	776 (46)
PICU admission	76 (0.2)	79 (1.3)	< 0.001	37 (0.9)	42 (2.5)
ILSI	385 (1.2)	253 (4.3)	< 0.001	121 (2.9)	132 (7.9)

Missing values: general patient characteristics: < 7%. Vital signs: 9–23%. NICE alarming signs 1–18%

*ED* emergency department, *GP* general practitioner, *PEWS* Paediatric Early Warning Score, *NICE* National Institute for Health and Care Excellence, *PICU* paediatric intensive care unit, *ILSI* immediate life-saving interventions

^a^Comorbidity: a chronic underlying condition that is expected to last at least 1 year. Complex comorbidity: a chronic condition in ≥ 2 body systems or malignancy or immunocompromised patients

^b^According to APLS cut-off values by age

### Patient characteristics

Patients with comorbidities were older (median age 3.7 years versus 2.6 years, *p* < 0.001) and more often were male (59% versus 54%, *p* < 0.001) than children without comorbidities.

Patients with comorbidities more often presented with a fever duration of less than 24 h (44% versus 35%, *p* < 0.001), more often were referred by a specialist (22% versus 8.3% (*p* < 0.001), and more often had a high triage urgency (53% versus 32%, *p* < 0.001), abnormal vital signs, or an ED-PEWS of 15 or higher (22% versus 12%, *p* < 0.001).

All differences were more pronounced in children with complex comorbidities (Table 2).

Furthermore, they more often were described as ill appearing (range according to comorbidity: 18–25%), had increased work of breathing (range 6.2–31%), or presented with neurological signs or symptoms (range 3.8–18%, Table 2; Appendix 4).

### Clinical management

In children with comorbidities, diagnostic tests such as general blood tests (aOR 2.0, 95 CI 1.9–2.2), CRP (aOR 2.0, 95% CI 1.8–2.1), blood cultures (aOR 3.0, 95% CI 2.7–3.3), and imaging (aOR 1.6, 95% CI 1.5–1.7) were performed more often after correcting for general patient characteristics. Furthermore, test results, such as CRP, and blood cultures (aOR 2.3, 95% CI 1.6–3.3) were more often abnormal (Table 3).

**Table 3:** Adjusted odds ratios for children with comorbidity for diagnostic tests, therapy, disposition, and final diagnosis

	**Multivariable**^**a**^
**Diagnostic tests**	
Any blood test	2.0 (1.9–2.2)
CRP performed	2.0 (1.8–2.1)
CRP > 60 mg/l	1.4 (1.3–1.6)
Imaging	1.6 (1.5–1.7)
Blood cultures	3.0 (2.7–3.3)
Blood cultures positive	2.3 (1.6–3.3)
Extensive testing^b^	1.6 (1.4–1.8)
**Therapy**	
ILSI	2.7 (2.2–3.3)
Oxygen therapy	4.9 (4.2–5.7)
Any antibiotics	1.6 (1.5–1.7)
Intravenous antibiotics	2.3 (2.1–2.5)
**Disposition**	
Any admission	2.2 (2.1–2.4)
PICU admission	5.5 (3.8–7.9)
Admission with an intervention	2.2 (2.0–2.4)

Children without comorbidity used as reference

*CRP* C-reactive protein, *ILSI* immediate life-saving interventions, *PICU* paediatric intensive care unit

^a^Adjusted for ED, age, sex, duration of fever, previous medical care, time of arrival

^b^Extensive testing was defined as three different types of tests; children that underwent a lumbar puncture were also scored as extensive testing

Regarding therapy, children with comorbidities more often required immediate life-saving interventions (aOR 2.7, 95 CI 2.2–3.3) and oxygen (aOR 4.9, 4.2–5.7) and were treated more frequently with intravenous antibiotics (aOR 2.3, 95% CI 2.1–2.5; Table 3). Children with comorbidities were admitted more often to the general ward (aOR 2.2, 95% CI 2.1–2.4) as well as the PICU (aOR 5.5, 95% 3.8–7.9; Table 3).

Children with a history of malignancy/immunodeficiency (aOR 5.8, 95% CI 4.8–7.0) or neurologic problems/psychomotor delay (aOR 2.9, 95% CI 2.5–3.3) were most often admitted, while children with a history of neurologic/psychomotor delay (aOR 9.7, 95% CI 6.1–15.5), children with a history of pulmonary disease (aOR 8.8, 95% CI 5.2–14.8), or children with a history of prematurity (8.1, 95% CI 4.4–14.7) were most often admitted to the PICU. Children with a history of neurologic disease/psychomotor delay (aOR 5.3, 95% CI 4.1–6.9), pulmonary disease (aOR 3.0, 95% CI 2.1–4.3), or cardiac disease (aOR 2.7, 95% CI 1.7–4.5) most often required immediate life-saving interventions (Appendix 5).

### Focus and presumed cause of infection

**Fig. 1:**
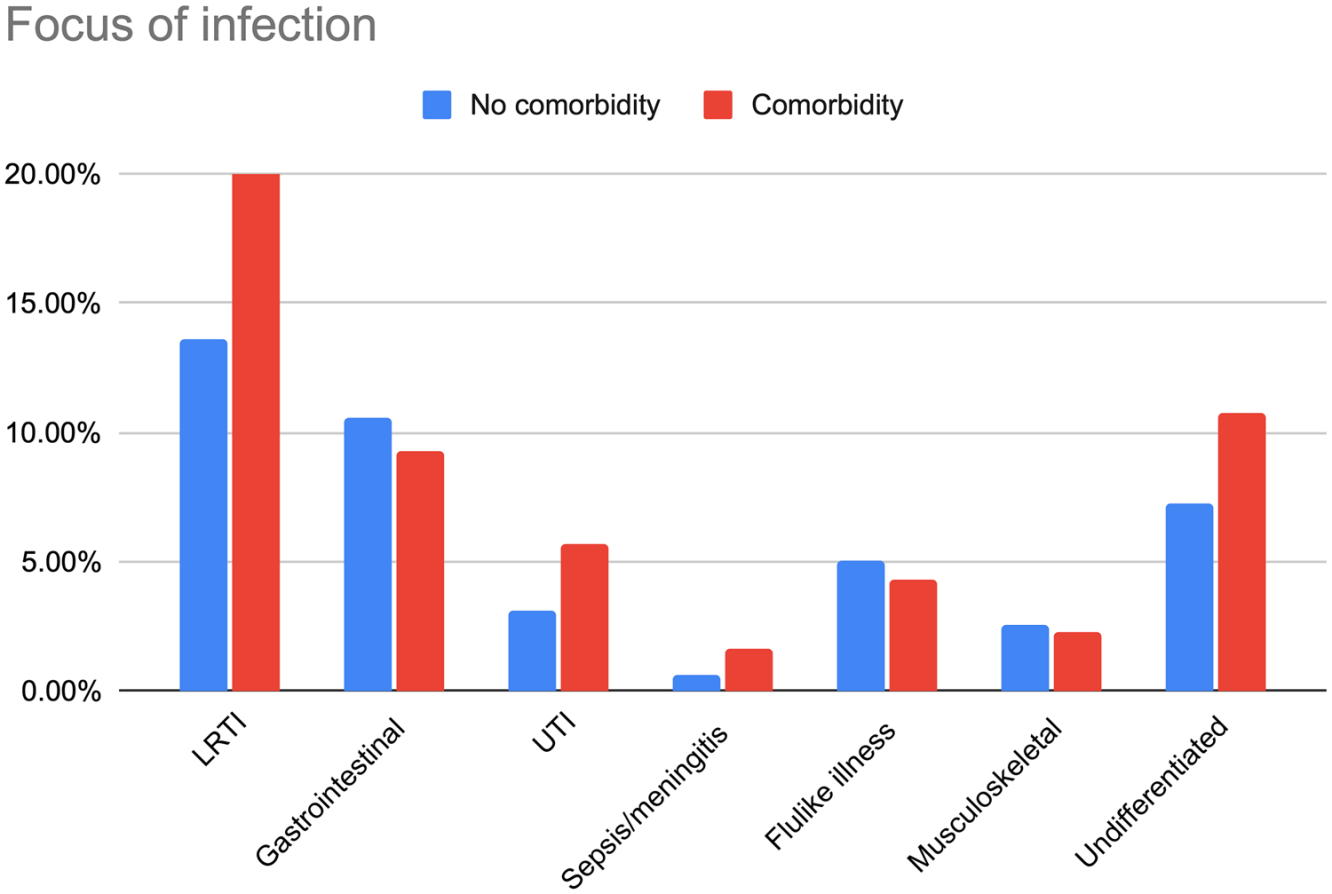
Focus of infection in children with and without comorbidity. Data shown as percentages within the groups of children with and without comorbidity. LRTI = lower respiratory tract infection; gastro-intestinal = gastro-intestinal and surgical abdomen; UTI = urinary tract infection, exanthems = exanthems and flulike illness; musculoskeletal = soft-tissue, skin and musculoskeletal infection. URTI (not shown in graphic) = upper respiratory tract infection: without comorbidity 54.5%, with comorbidity 41.2%

Most common types of infections in almost all subgroups of comorbidity were upper respiratory tract infections, lower respiratory tract infections, and undifferentiated fever (Appendix 6). Children with comorbidities were more often diagnosed with lower respiratory tract (20.4 versus 13.6, *p* < 0.001; Fig. 1), undifferentiated fever (10.8 versus 7.3, *p* < 0.001), and sepsis/meningitis (1.6 versus 0.6%, *p* < 0.001), while presentations for upper respiratory tract infections (41 versus 55%, *p* < 0.001) were less common.

After correcting for general patient characteristics, patients with comorbidities more often were categorized as having a serious bacterial infection (aOR 1.8, 95% CI 1.7–2.0) and invasive bacterial infections (sepsis/meningitis) in particular (aOR 4.6, 95% CI 3.2–6.7). Children most at risk for sepsis/meningitis were children with a history of malignancy/immunodeficiency (aOR 14.5, 95% CI 8.5–24.8), neurologic disease/psychomotor delay (aOR 4.6, 95% CI 2.6–8.1), or prematurity (aOR 4.5, 95% CI 2.2–9.2; Appendix 5). These results were similar when only including children with culture-proven sepsis/meningitis (Appendix 7). In our study population, *Escherichia coli*, *Staphylococcus aureus*, coagulase-negative *Staphylococci*, and *Streptococcus pneumoniae* were the most common pathogens found in children with comorbidities, while *Neisseria meningitidis*, group B *Streptococcus, Streptococcus pneumoniae*, and *Escherichia* coli were the most common pathogens in febrile children without comorbidities.

## Discussion

### Main findings

Children with comorbidities, with 16%, form a substantial part of the paediatric ED population. Our data show that children with comorbidities in general are more ill upon presentation than children without comorbidities, as they more often have abnormal vital signs, and a high ED-Paediatric Early Warning Score. In contrast with this, in our study, children with comorbidity presented with a shorter duration of fever, possibly due to awareness of a higher risk for serious illness. They more often present to the ED with common diseases such as lower respiratory tract infections as well as invasive bacterial infections such as sepsis/meningitis. While they are managed differently, with higher rates of resource use such as blood tests, admission, intravenous antibiotics, and rates of serious interventions (immediate life-saving interventions, PICU admission), this seems adequate as they are diagnosed with serious bacterial infections more often. Secondly, our data show that children with comorbidities form a heterogeneous group, with different types of comorbidities requiring a distinct pattern of management. As expected, children with malignancy/immunodeficiency more often had serious bacterial infections and were treated with intravenous antibiotics, and children with psychomotor delay/neurological disease most often required immediate life-saving interventions and PICU admission.

### Findings in relation to previous literature

Studies on the prevalence of chronic comorbidities in the paediatric population are scarce and many articles base their numbers on US studies that took place more than a decade ago [26–28]. Using these numbers might underestimate the current prevalence and health care burden of children with comorbidities [1].

Furthermore, comparing studies is not straightforward due to the fact that studies differ in study methods (e.g. self-reported comorbidity versus hospital data versus national registries), definitions used and whether mental health is included in the definition [19, 20, 28–31].

These differences might explain the large variation that is found in the prevalence of these children in Europe, which varies between 10 and 40% [29, 31, 32].

However, there is convincing data that regardless of the definition used, children with comorbidity have higher health care utilization and an increased risk of in-hospital mortality [20, 28, 33–35].

For example, a recent European study showed that depending on the setting, between 10 and 38% of all ED visits were comprised by children with comorbidities [33].

Furthermore, several studies showed that between 40 and 75% of cases of childhood mortality were comprised by children with comorbidities [28, 34, 35].

Our study is in line with previous studies that found increased resource use in children with comorbidities and an increased risk of serious bacterial infections [4, 9, 14, 36]. With our study, we aimed to provide a more detailed overview of these children and identify children at risk for serious illness.

### Implications for clinical practice and research

Our data demonstrate how children with comorbidities form a fragile patient population. Clinicians should be aware of the increased risk for serious bacterial infections and PICU admission when evaluating febrile children with comorbidities at the ED.

Given their increasing numbers on one hand and increased risk for serious bacterial infections on the other hand, it is imperative that they are not being left out of studies or guidelines. Our study provides insight on which specific subgroups are specifically at risk for serious bacterial infections and interventions such as immediate life-saving interventions or PICU admission. Children most at risk for sepsis/meningitis were children with a history of malignancy/immunodeficiency, prematurity or neurologic disease/psychomotor delay. These data could be used to maintain a lower threshold for diagnostic tests, and start antibiotic therapy based on the combination of clinical assessment and diagnostic test results, children with a history of neurologic/psychomotor delay, pulmonary disease, cardiac disease, or prematurity most often required PICU admission or immediate life-saving interventions. Further research should identify which subgroups of children are most at risk for serious illness and provide detailed information on the disease course. This information should ideally be used to improve early recognition and interventions in order to improve the outcome of these children.

As most, though not all [37], studies predicting serious bacterial infections in febrile children have excluded children with comorbidities, future studies should focus on validating existing clinical prediction rules for this population, and if necessary, develop guidelines and prediction rules specifically targeted to this population. Our data show that, although overall, children with comorbidity have an increased risk for serious bacterial infections; this is not true for all subgroups of comorbidity. This data can be used to target antibiotic therapy.

Lastly, future research would benefit from the use of a uniform classification of children with comorbidities that can be used to provide an overview of the prevalence and resource use in these children at all levels of care. Furthermore, using a uniform classification can facilitate the comparison of different studies.

### Strengths and limitations

To our knowledge, our study is the first to include a large multicenter cohort of febrile children with different types of comorbidities and includes detailed information on presenting signs and symptoms, management, diagnostic test results, and cause of infection.

Data were collected year-round and included different EDs with different rates of children with comorbidities, which largely increases the generalizability of the results [16, 38].

Furthermore, we have included a large number of children with serious and invasive bacterial infections, which was determined by a uniformly applied and validated flowchart [16].

Using routinely collected data has its limitations. However, to ensure data quality and completeness, all study sites were extensively trained regarding the accurate documentation of patient characteristics and quality checks were performed regularly. Missing data were limited, and its effects were further reduced by using multiple imputation for missing values [39].

A second limitation is that in some settings, children that are likely to be admitted, for example due to a high risk for serious bacterial infections, such as children with febrile neutropenia, are sometimes seen at the ward directly and bypass the ED [40]. Furthermore, data were only collected at the ED and not at primary care facilities. Therefore, the patients included in our study might not represent the complete group of febrile children with comorbidity. Furthermore, although comorbidity was grouped by body system, these groups could still be heterogeneous. However, heterogeneity was reduced by further analysing children by level of complexity.

Lastly, although this study provided detailed information by children with comorbidity by body system, we did not study resource use and risk of serious illness for specific diagnoses.

## Conclusion

Our data show that children with comorbidity form important part of the paediatric ED population. Febrile children with comorbidities in general are more ill with a shorter duration of symptoms, more frequently have abnormal test results, more often require admission and PICU admission and life-saving interventions, and more often are diagnosed with serious and invasive bacterial infections.

## Supplementary Information

Below is the link to the electronic supplementary material.

Supplementary file1 (PDF 656 kb)

## Data Availability

Individual participant data that underlie the results reported in this article, including a data dictionary, will be made available after de-identification to researchers who provide a methodologically sound proposal. Proposals should be directed to d.borensztajn@erasmusmc.nl. To gain access, data requestors will need to sign a data access agreement.

## Abbreviations

aOR: Adjusted odds ratio
CRP: C-reactive protein
ED: Emergency department
MOFICHE: Management and Outcome of Fever in children in Europe
NICE: National Institute for Health and Care Excellence
ILSI: Immediate life-saving interventions
PERFORM: Personalized Risk assessment in Febrile illness to Optimize Real-life Management across the European Union
PEWS: Paediatric Early Warning Score
PICU: Paediatric Intensive Care Unit

## Declarations

### Disclaimer

The views expressed are those of the author(s) and not necessarily those of the NHS, the NIHR, or the Department of Health.
