# Supplementary material for: Febrile children with comorbidities at the emergency department — a multicentre observational study
Source: Eur J Pediatr. 2022 Jul 7;181(9):3491–500. doi: 10.1007/s00431-022-04552-2 (PMC9395458; doi:10.1007/s00431-022-04552-2)
Supplement: Supplementary file 1 — Supplementary file1 (PDF 656 kb) [file 431_2022_4552_MOESM1_ESM.pdf]

Title: Febrile children with comorbidities at the ED – A multicentre observational study.

European Journal of Pediatrics

Dorine M. Borensztajn, MD, MSc[1] Nienke N. Hagedoorn, MD, Prof. Enitan D. Carrol, MD, PhD,[2,3,4] Ulrich von Both, MD, FRCPCH[5,6] Prof. Marieke Emonts, MD, PhD,[8,9,10] Michiel van der Flier, MD, PhD,[11,12] Prof. Ronald de Groot, MD, PhD,[13,14] Jethro Herberg, MD, PhD[15] Benno Kohlmaier, MD,[16] Prof. Michael Levin, MD, PhD,[15] Emma Lim, MD[8], Ian K. Maconochie, MD, PhD,[15] Prof. Federico Martinon-Torres, MD, PhD[17], Ruud G. Nijman, MD, PhD,[15] Marko Pokorn, MD, PhD,[18] Irene Rivero-Calle, MD, PhD,[17] prof. Maria Tsolia, MD, PhD,[19] Fabian J.S. van der Velden, MD, MSc[8,9], Clementien Vermont, MD, PhD,[20] Dace Zavadska, MD, PhD[21] Prof. Werner Zenz, MD, PhD,[16] Joany M. Zachariasse, MD, PhD,[1] Prof. Henriette A. Moll, MD, PhD.[1]

On behalf of PERFORM consortium: Personalised Risk assessment in febrile children to optimise Real-life Management across the European Union.

Author affiliations:

1. Erasmus MC-Sophia Children's Hospital, Department of General Paediatrics, Rotterdam, the Netherlands.
2. University of Liverpool, Institute of Infection, Veterinary and Ecological Sciences, Liverpool, United Kingdom.
3. Department of Infectious Diseases, Alder Hey Children's NHS Foundation Trust, Liverpool, UK
4. Liverpool Health Partners, Liverpool, UK
5. Division of Paediatric Infectious Diseases, Dr. von Hauner Children's Hospital, University hospital, Ludwig-Maximilians-University (LMU), Munich, Germany.
6. German Centre for Infection Research, DZIF, Partner Site Munich, Munich, Germany
7. London School of Hygiene and Tropical Medicine, Faculty of Tropical and Infectious Disease, London, United Kingdom.

8. Great North Children's Hospital, Paediatric Immunology, Infectious Diseases & Allergy, Newcastle upon Tyne Hospitals NHS Foundation Trust, Newcastle upon Tyne, United Kingdom.
9. Translational and Clinical Research Institute, Newcastle University, Newcastle upon Tyne, United Kingdom
10. NIHR Newcastle Biomedical Research Centre based at Newcastle upon Tyne Hospitals NHS Trust and Newcastle University, Newcastle upon Tyne, United Kingdom
11. Paediatric infectious diseases and immunology, Amalia Children's Hospital, Radboudumc, Nijmegen, The Netherlands.
12. Paediatric infectious diseases and immunology, Wilhelmina Children's Hospital, University Medical Centre Utrecht, Utrecht, The Netherlands.
13. Section Paediatric Infectious Diseases, Laboratory of Medical Immunology, Department of Laboratory Medicine, Radboud Institute for Molecular Sciences, Radboud University Medical Centre, Nijmegen, the Netherlands
14. Radboud Center for Infectious Diseases, Radboudumc, 6525 GA, Nijmegen, The Netherlands.
15. Imperial College of Science, Technology and Medicine, Section of Paediatric Infectious Diseases, London, United Kingdom.
16. Medical University of Graz, Department of General Paediatrics, Graz, Austria.
17. Hospital Clínico Universitario de Santiago de Compostela, Genetics, Vaccines, Infections and Pediatrics Research group (GENVIP), Santiago de Compostela, Spain
18. University Medical Centre Ljubljana, Univerzitetni Klinični Center, Department of Infectious Diseases, Ljubljana, Slovenia.
19. National and Kapodistrian University of Athens, Second Department of Paediatrics, P. and A. Kyriakou Children's Hospital, Athens, Greece.
20. Erasmus MC-Sophia Children's Hospital, Department of Pediatric infectious diseases & immunology, Rotterdam, the Netherlands.
21. Rīgas Stradiņa universitāte, Children clinical university hospital, Department of Pediatrics, Riga, Latvia

Corresponding author:

Dorine M. Borensztajn

Department of General Paediatrics, Erasmus MC – Sophia Children’s Hospital

Appendix 1: Participating EDs

Hospital	Country, city	Hospital type	Total annual paediatric ED visits	Total period of inclusion	Period of inclusion per month	Number of patients included
Medizinische Universität Graz	Austria, Graz	University	10,000-30,000	1-1-2017 – 31-12-2018	10 days	2243
Dr. von Hauner Children's Hospital	Germany, Munich	Teaching	10,000-30,000	1-1-2017 – 31-12-2018	1 week	1175
P. and A. Kyriakou Children's Hospital	Greece, Athens	University	>30,000	1-1-2017 – 1-5-2018	1-2 weeks	4549
Children clinical university hospital	Latvia, Riga	Teaching	>30,000	1-1-2017 – 31-12-2018	All	9000
Univerzitetni Klinični Center	Slovenia, Ljubljana	University	<10,000	1-1-2017 – 31-12-2018	All	3659
Hospital Clínico Universitario	Spain, Santiago de Compostela	University	>30,000	1-1-2017 – 1-5-2018	1-2 weeks	3877
Erasmus MC-Sophia Children's Hospital	The Netherlands, Rotterdam	University	<10,000	1-1-2017 – 1-4-2018	All	1681
RadboudUMC	The Netherlands, Nijmegen	University	<10,000	1-1-2017 – 1-4-2018	All	676
Canisius Wilhelmina Ziekenhuis	The Netherlands, Nijmegen	Teaching	<10,000	1-1-2017 – 31-12-2018	2 weeks	415
Alder Hey Children's Hospital	United Kingdom, Liverpool	Teaching	>30,000	1-1-2017 – 31-12-2018	1 week	1624
St. Mary's Hospital	United Kingdom, London	University	10,000-30,000	1-1-2017 – 31-12-2018	All	5714
Great North Children's Hospital	United Kingdom, Newcastle upon Tyne	University	>30,000	1-4-2017 – 1-4-2018	2 weeks	3870

Appendix 2. Immediate life-saving interventions

1. Airway and breathing support, including intubation or emergent noninvasive positive pressure ventilation.
2. Electrical therapy, including defibrillation, emergent cardioversion, or external pacing.
3. Procedures, including chest needle decompression, pericardiocentesis, or open thoracotomy.
4. Hemodynamic support, including significant intravenous fluid resuscitation in the setting of hypotension, blood administration, or control of major bleeding.
5. Emergency medications, including naloxone, dextrose, atropine, adenosine, epinephrine, or vasopressors

Appendix 3 - Categorization of presumed cause of infection

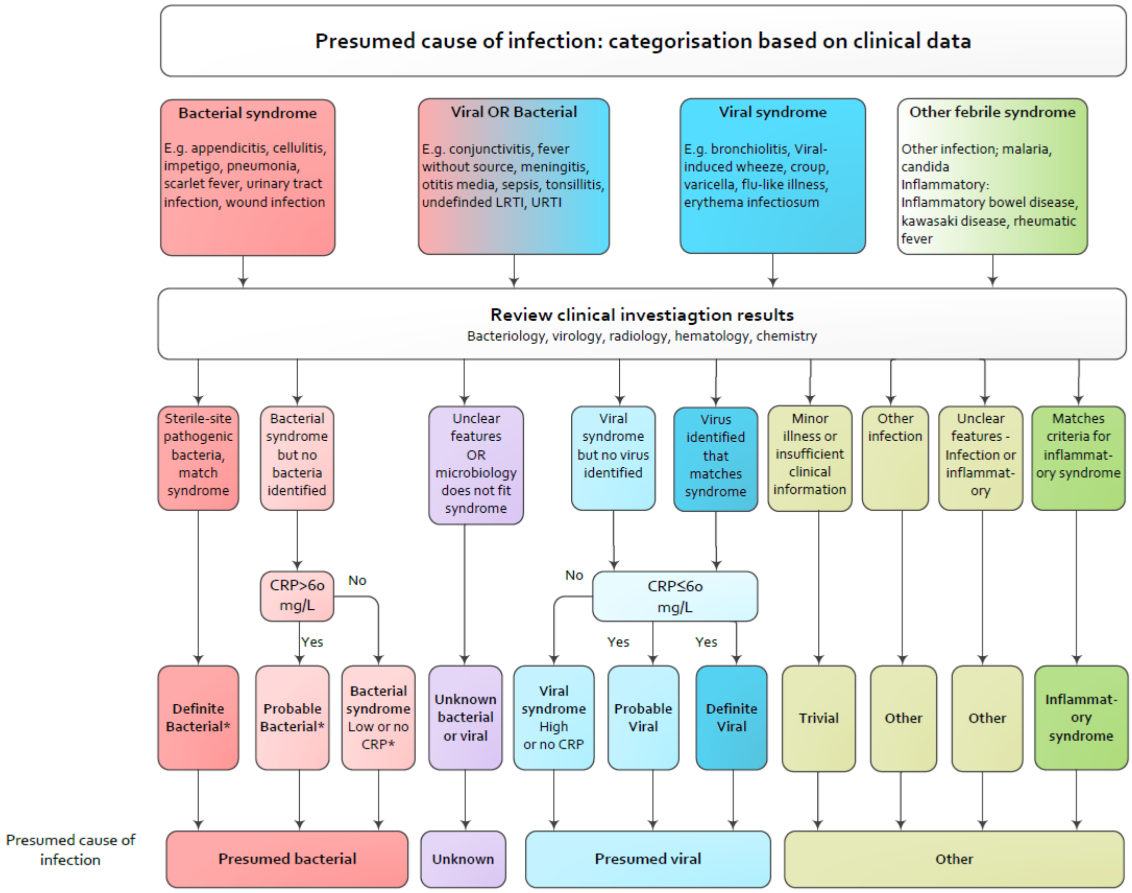

Appendix 4: NICE alarming signs for children with different types of comorbidities.

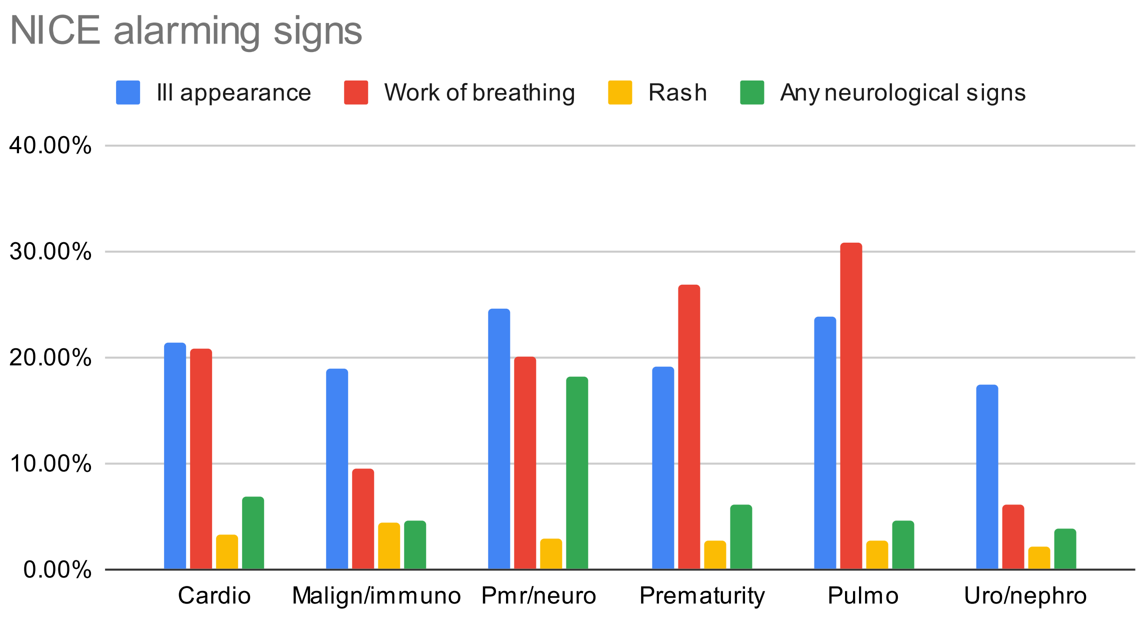

Appendix 5: Adjusted odds ratios for children with specific types of comorbidities for diagnostic tests, therapy, disposition and final diagnosis compared to children without comorbidities. [#]

	Cardiac	Malignancy-immunodeficiency	Psychomotor delay-neurological	Prematurity	Pulmonary	Urology/ nephrology
**CRP > 60**	1.5 (1.1-2.1)	2.5 (2.0-3.2)	1.6 (1.3-1.9)	1.1 (0.8-1.5)	0.9 (0.7-1.1)	2.1 (1.6-2.8)
**Positive blood cultures**	1.5 (0.6-4.0)	3.4 (2.0-5.6)	1.8 (1.0-3.3)	1.4 (0.6-3.3)	2.6 (1.2-5.6)	2.0 (0.8-5.0)
**ILSI**	2.7 (1.7-4.5)	2.3 (1.4-3.8)	5.3 (4.1-6.9)	2.2 (1.4-3.3)	3.0 (2.1-4.3)	1.3 (0.7-2.5)
**IV antibiotics**	1.9 (1.4-2.5)	9.3 (7.7-11.2)	2.6 (2.2-3.0)	1.6 (1.2-2.0)	1.4 (1.1-1.7)	2.4 (1.9-3.0)
**Any admission**	2.1 (1.7-2.6)	5.8 (4.8-7.0)	2.9 (2.5-3.3)	2.1 (1.8-2.5)	2.1 (1.9-2.5)	1.8 (1.5-2.2)
**PICU admission**	6.9 (3.2-14.7)	6.2 (2.8-13.6)	9.7 (6.1-15.5)	8.1 (4.4-14.7)	8.8 (5.2-14.8)	0.0 (0.0-0.0)
**Sepsis/meningitis**	3.0 (1.1-8.4)	14.5 (8.5-24.8)	4.6 (2.6-8.1)	4.5 (2.2-9.2)	2.2 (1.0-5.0)	2.6 (0.9-7.3)

Children without comorbidity used as reference.
Adjusted for ED, age, sex, duration of fever, previous medical care, time of arrival.

Appendix 6: focus of infection for children with different types of comorbidities.

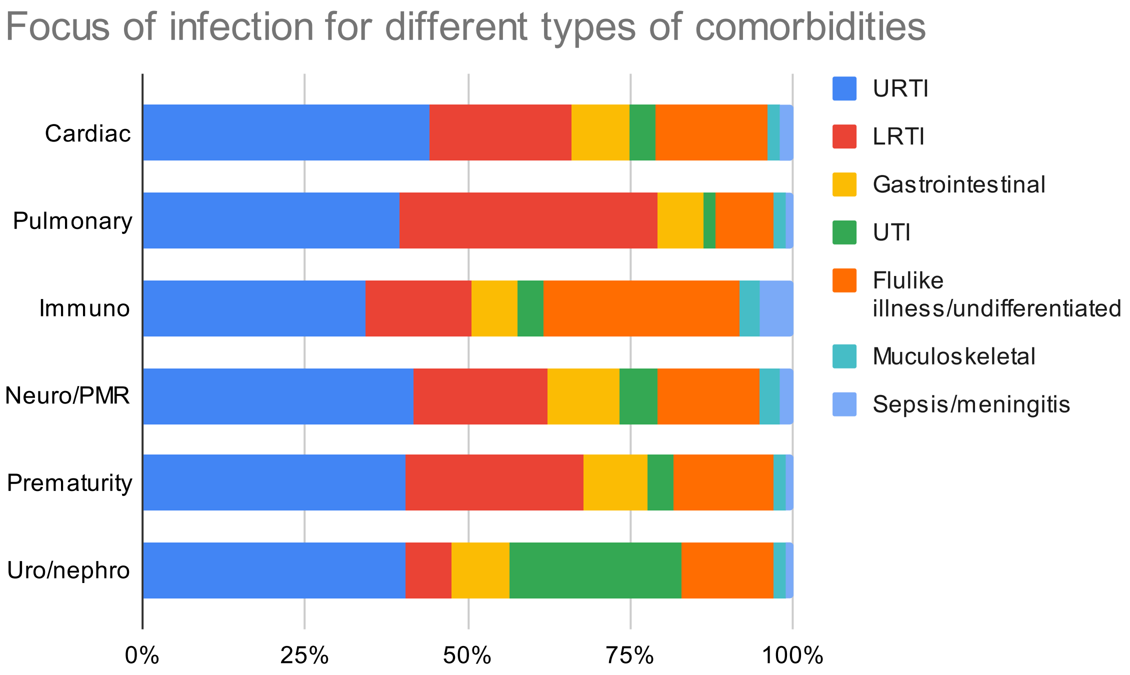

Appendix 7: Pathogens found in children with sepsis/meningitis with (N=45) and without comorbidity (N=25).

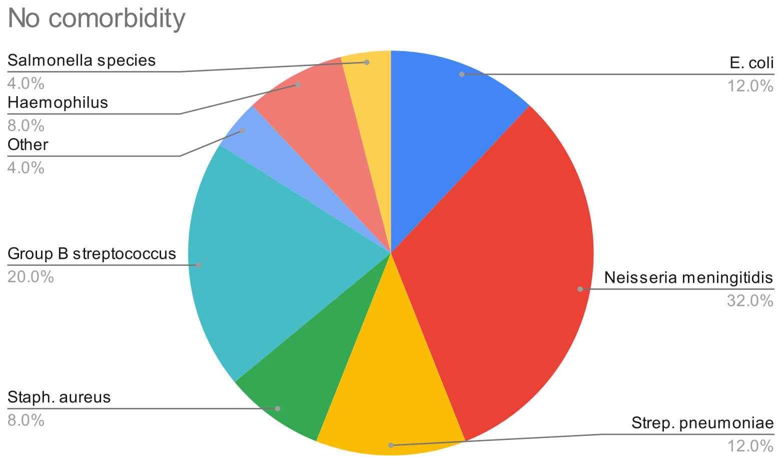

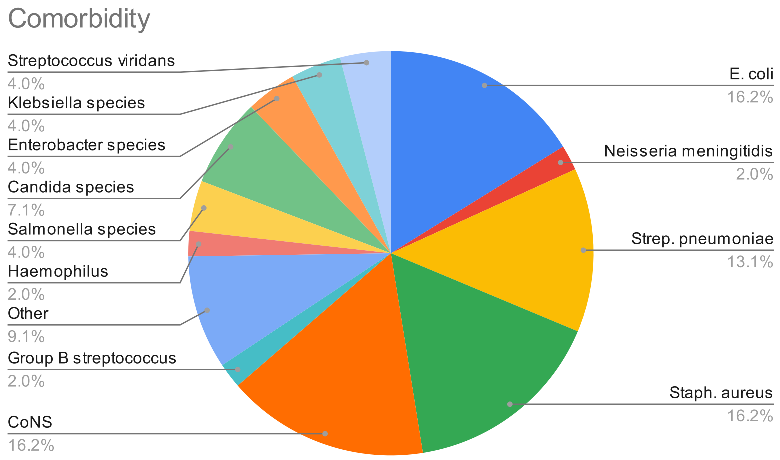

CoNS = Coagulase-negative staphylococci. CoNS were considered contaminants in children with comorbidity.
